# Transoral Circular Stapler for Cervical Anastomosis During Esophagectomy

**DOI:** 10.1016/j.atssr.2025.10.002

**Published:** 2025-11-05

**Authors:** Angel Doño, Jane Zhao, Brenden Sheridan, Calvin Jung, Jenna Davis, Jasmine Ngo, Ganpat Valaulikar, Thomas Ng

**Affiliations:** Division of Thoracic Surgery, Department of Surgery, University of Tennessee Health Science Center, College of Medicine, Memphis, Tennessee

## Abstract

**Background:**

This report describes a transoral circular stapling technique for cervical anastomosis during esophagectomy.

**Methods:**

After the gastric conduit is delivered into the neck, the plastic guide of the transoral circular anvil is passed through the mouth and retrieved through a small opening made in the center of the linear cervical esophagus staple line, facilitating the passage of the circular anvil through the mouth into the cervical esophagus. The stapler handle is introduced into the gastric conduit through a gastrotomy created in the excess stomach, the spike is advanced, puncturing the greater curvature of the more distal conduit, connected to the circular anvil, and fired. The excess gastric conduit, including the gastrotomy site, is resected using a linear stapler.

**Results:**

Ten consecutive patients (3 women, 7 men) underwent cervical anastomoses (6 transhiatal and 4 three-incision esophagectomy). Median age was 62 years, median body mass index was 24 kg/m^2^, median blood loss was 350 mL, and median operative time was 285 minutes. Pathology included 2 patients with extensive high-grade dysplasia, 1 with refractory reflux-induced stricture, and 7 with malignancy (adenocarcinoma in 6 and squamous cell carcinoma in 1). All patients had complete esophageal and gastric donuts and were without leak during intraoperative endoscopy. There were no clinical or radiographic anastomotic leaks or strictures and no mortality at 90 days.

**Conclusions:**

Cervical anastomosis with the transoral circular stapler is feasible. The technique is reproducible and results in a consistent and widely patent anastomosis with low leak and stricture rates. Larger studies are warranted to confirm our results.

**In Short**

▪ Cervical anastomosis with the transoral circular stapler is feasible.
▪ The technique is reproducible and results in a consistent and widely patent anastomosis with low leak and stricture rates.

After esophagectomy, cervical esophagogastric anastomosis is typically performed with handsewn or a partial linear stapling technique.^1^^,^^2^ In this report, we describe a total stapled cervical esophagogastric anastomosis using the transoral circular stapler. The technique and outcomes are described in detail.

## Patients and Methods

### Patient Cohort

Consecutive patients who underwent cervical esophagogastric anastomosis after transhiatal or 3-incision esophagectomy were evaluated. Details of the anastomotic technique, patient demographics, intraoperative data, and postoperative outcomes are reported. The study was approved by the University of Tennessee Health Science Center Institutional Review Board (24-10213-XP). Consent was waived because this was a retrospective study.

### Surgical Technique

#### Esophagectomy Approach

For the 3-incision esophagectomy (McKeown), thoracic dissection is performed under direct vision through a right thoracotomy, fully mobilizing the thoracic esophagus from the diaphragmatic hiatus to the thoracic inlet. The azygous vein is transected, and paraesophageal and subcarinal nodes are dissected. Radical en bloc resection is not routinely performed. After the thoracic phase is completed, the thoracotomy is closed, and the patient is repositioned supine for left cervical incision and laparotomy.

For transhiatal esophagectomy, the patient is placed supine for left cervical incision and laparotomy. Thoracic esophageal dissection is performed bluntly using the surgeon’s hand, with the inferior dissection carried out transhiatally from the abdomen and superior dissection from the left cervical incision. Pyloroplasty and feeding jejunostomy are performed routinely in both approaches.

#### Gastric Mobilization

**Figure 1:**
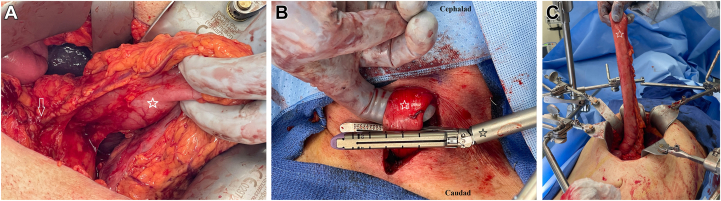
Gastric conduit mobilization and creation. (A) Maximum gastric (star) mobilization involves division of all the epiploic branches of the right gastroepiploic artery down to its origin (arrow). (B) In the left neck, the cervical esophagus (white star) is transected 3 to 4 cm below the cricopharyngeal muscle using the liner endostapler (black star). (C) Stepwise application of the linear endostapler during gastric tube formation straightens the natural “C” shape of the stomach to create more conduit length (star); this, along with maximum gastric mobilization, allows for excess conduit length when delivered into the neck, which is essential for this anastomotic technique.

Adequate gastric mobilization is essential, because excess conduit length is required for this anastomotic technique. Mobilization is performed using a combination of a linear vascular endostapler and a bipolar vessel sealing device. The left gastric artery, left gastroepiploic artery, short gastric arteries, and all the epiploic branches of the right gastroepiploic artery are divided to its origin, thereby eliminating tethering of the conduit by the colon (Figure 1A). A generous Kocher maneuver is then performed, elevating the duodenum from the retroperitoneum.

#### Gastric Conduit Formation

Through the left cervical incision, the esophagus is transected 3 to 4 cm below the cricopharyngeal muscle with the liner endostapler (Figure 1B). The esophagus is then delivered into the abdomen, and a 3- to 4-cm-wide gastric tube is created using serial firings of the linear endostapler. Stepwise application of the stapler straightens the natural “C” shape of the stomach to create more conduit length. This, along with maximum gastric mobilization, as described in the previous section, allows for not only adequate reach to the cervical incision but also excess conduit length when delivered into the neck, which is essential for this anastomotic technique (Figure 1C).

**Figure 2:**
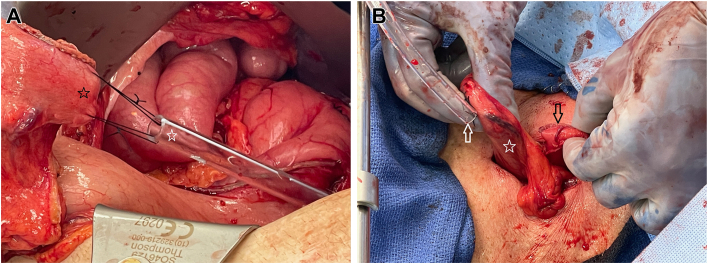
Delivery of the gastric conduit into the left neck. (A) A 28F chest tube (white star) is then passed from the left cervical incision directed inferiorly into the mediastinum, through the hiatus and into the abdomen, where it is loosely stitched to the tip of the gastric conduit (black star). (B) The chest tube (white arrow) aides in passing the conduit through the hiatus, up the mediastinum, and into the cervical incision. With proper gastric mobilization and tube formation, there should be abundant gastric conduit length (star) in the neck for the anastomosis with the esophagus (black arrow).

The specimen of esophagus and stomach is removed from the field. A 28F chest tube is passed from the left cervical incision and directed inferiorly through the mediastinum and hiatus into the abdomen. The chest tube is loosely sutured to the tip of the gastric conduit (Figure 2A), facilitating its passage through the hiatus and mediastinum into the neck. With proper mobilization and tube formation, ample conduit length is available once delivered into the neck (Figure 2B).

#### Cervical Esophagogastric Anastomosis

**Figure 3:**
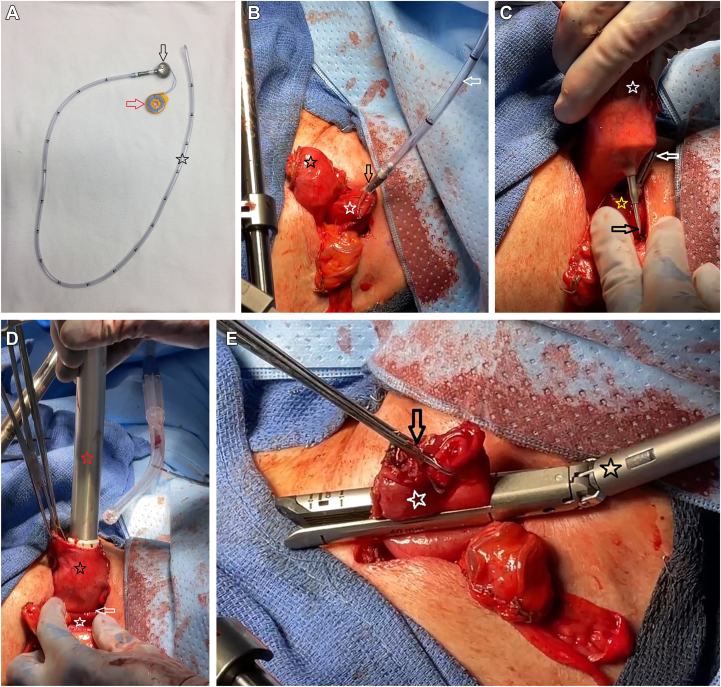
Cervical esophagogastric anastomosis using the transoral circular stapler. (A) The 25-mm circular anvil (black arrow) is attached to a plastic guide (star) that guides the anvil through the mouth into the remaining cervical esophagus for the anastomosis. If necessary, the circular anvil can be retrieved with the retrieval string (red arrow). (B) The plastic guide is retrieved into the operative field of the left neck through a small opening in the middle of the cervical esophageal staple line. Then, antegrade traction on the plastic guide (white arrow) allows the circular anvil (black arrow) to pass through the mouth and be positioned into the cervical esophagus (white star), ready for anastomosis with the gastric conduit (black star). (C). The circular staple handle is placed into the stomach conduit through a gastrotomy made in the excess portion of the conduit (white star), and the spike (black arrow) is advanced to puncture the greater curvature of the more distal aspect of the conduit. The anvil (white arrow) within the esophagus (yellow star) will be connected to the spike of the staple handle for the anastomosis. (D) The circular staple handle (red star) is then tightened to securely approximate the cervical esophagus (white star) with the gastric conduit (black star) for the anastomosis (arrow) and fired. (E) The excess gastric conduit (white star) is then removed using the linear endostapler (black star), removing the gastrotomy site (arrow) where the circular staple handle was inserted.

The plastic guide, which is attached to the 25-mm transoral circular anvil (The OrVil, Covidien) (Figure 3A), is passed through the patient’s mouth into the cervical esophagus. The plastic guide is retrieved into the operative field through a small opening cut in the center of the cervical esophageal staple line. Antegrade traction on the guide advances the circular anvil through the mouth to be positioned into the cervical esophagus (Figure 3B). The guide is then detached, leaving the anvil ready for connection to the stapler handle (Figure 3C). The anastomosis site on the gastric conduit is chosen along the greater curvature at a point that lays without tension adjacent to the transected esophagus. This is typically a more distal aspect of the conduit, because excess length is available after mobilization.

The 25-mm end-to-end anastomosis circular staple handle (EEA 25-mm XL, Covidien), is introduced into the gastric conduit through a gastrotomy created in the proximal-most aspect of the conduit, which represents an area of excess stomach (Figure 3C). The spike of the stapler handle is advanced to pierce the greater curvature of conduit at the predetermined anastomotic site (Figure 3C) and is connected to the circular anvil. The staple handle is tightened, approximating the cervical esophagus with the gastric conduit and then fired (Figure 3D). The stapler handle is removed, and 2 complete anastomotic donuts are retrieved and sent to pathology.

**Figure 4:**
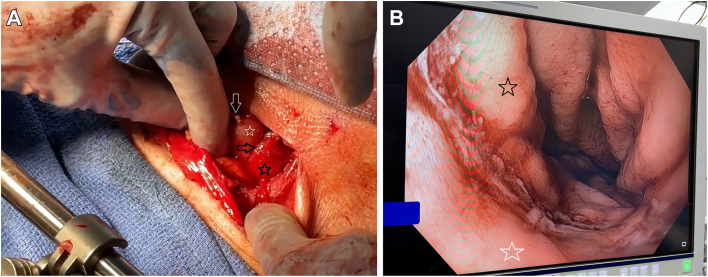
Appearance of the anastomosis. (A) This final conduit linear staple line (white arrow) should be away from the circular staple line (black arrow) of the esophago- (black star) gastric (white star) anastomosis, to prevent conduit ischemia between 2 close staple lines. (B) Upper endoscopy shows an intact, widely patent anastomosis, with healthy gastric conduit (black star) and esophageal (white star) mucosa.

The excess gastric conduit containing the gastrotomy is then resected with a linear endostapler, thereby also removing the stapler insertion site (Figure 3E). This technique avoids gastrotomy within the final conduit, preserves the submucosal blood supply, and prevents use of the proximal conduit tip (which is the most relative ischemic portion) for anastomosis. The final conduit linear staple line should be away from the circular anastomotic staple line to minimize the risk of ischemia between 2 close staple lines (Figure 4A). Upper endoscopy is performed to confirm an intact, widely patent anastomosis with healthy gastric conduit and esophageal mucosa as well as no leak upon insufflation (Figure 4B).

## Results

Cervical anastomosis with the transoral circular stapler was performed in 10 consecutive patients (3 women and 7 men), with 6 transhiatal, and 4 three-incision esophagectomies. Median age was 62 years (interquartile range, 57-70 years). Median body mass index was 24 kg/m^2^ (interquartile range, 22-35 kg/m^2^), median blood loss was 350 mL (interquartile range, 250-500 mL), and median operative time was 285 minutes (interquartile range, 255-345 minutes). Pathology revealed 2 patients with extensive high-grade dysplasia, 1 with refractory reflux stricture, and 7 with malignancy (adenocarcinoma in 6, squamous cell carcinoma in 1). Six patients received neoadjuvant chemoradiation for advanced disease; none received adjuvant therapy.

In all patients, complete esophageal and gastric donuts were retrieved, and intraoperative endoscopy confirmed a widely patent anastomosis without leak upon insufflation. A barium esophagram on postoperative day 6 in all patients showed no anastomotic leak detected. Four patients experienced postoperative complications, comprising 1 wound infection, 1 chylothorax, 1 urinary tract infection, and 1 with combined atrial fibrillation, pneumonia, and deep venous thrombosis. There were no clinical or radiographic anastomotic leaks, and no mortality was seen at 90 days. At a median follow-up time of 12 months, no patient had clinical evidence of stricture or required endoscopic dilation.

## Comment

Cervical anastomosis after esophagectomy is commonly performed by a handsewn or linear stapler technique, as described by Orringer and colleagues.^1^^,^^2^ Here, we report consecutive patients undergoing total stapled cervical anastomosis using a transoral circular stapler (Video 1). This technique has been described for thoracic esophagogastric anastomosis during Ivor Lewis esophagectomy.^3^ Although using the circular stapler for cervical anastomosis has been described,^4^ these involved placing the circular anvil into an open esophagus, followed by purse string closure, whereas we use a transoral technique for positioning the circular anvil into the cervical esophagus for the anastomosis.

We found this technique to be feasible, reproducible, and efficient, resulting in a consistently wide anastomosis with low leak and stricture rates. Larger studies are warranted to confirm our results and to compare this technique with other cervical anastomotic techniques.
